# Death of Dr. Quimby

**Published:** 1898-06

**Authors:** 


DEATH OF DR. QUIMBY.—In the death of Dr. Isaac Newton Quimby, of Jersey City, New Jersey, which occurred at his home in that city at 5:30 p. m., May 6, (ult.) the medical profession has sustained a very great loss; and the cause of temperance, and reform in criminal jurisprudence are robbed of one of their ablest and most gifted and zealous apostles.

Dr. Quimby was no ordinary man. He was a great man; and like all advanced thinkers—he was ahead of his generation. He was by some regarded as a crank;—many persons cannot diagnose genius,—he was no crank, but a thinker—who had the courage to speak his convictions. His work will live after him, tho', and be appreciated bye and bye. The seeds he has sown have fallen in good soil, we feel assured, and will bring forth fruit in due season.

Dr. Quimby was a self-made man. He began life as a farmer's boy, at the plow and the hoe; and by the force of his inherent genius climbed to the summit of fame in a crowded profession sparkling with stars of his own stamp and lustre; but few of whom equalled, and none outshone him.

Judge Clark Bell, of New York, the famous medico-legal scholar and writer, was one of the pall-bearers. Dr. Quimby's funeral was attended by distinguished scholars from many States. They appreciate the extent of the loss to science and humanity, and with the bereaved family they beg to share the grief. Dr. Quimby was 65 years of age. He died of pneumonia.

It was the writer's privilege to have met and known the doctor, and we feel that his loss is a personal one to us. Peace to his great soul in the beautiful Beyond!

